# Supplementary material for: OpenMM 7: Rapid development of high performance algorithms for molecular dynamics
Source: PLoS Comput Biol. 2017 Jul 26;13(7):e1005659. doi: 10.1371/journal.pcbi.1005659 (PMC5549999; doi:10.1371/journal.pcbi.1005659)
Supplement: S1 Source Code — (ZIP) [file pcbi.1005659.s002.zip › OpenMM-7.0.1-Source/docs/api-python/library.html]

Library Layer — OpenMM 7.0.1.dev-5e86c4f documentation


# Library Layer¶

## Core Objects¶

|  |  |
| --- | --- |
| `System` | This class represents a molecular system. |
| `Context` | A Context stores the complete state of a simulation. |
| `Platform` | A Platform defines an implementation of all the kernels needed to perform some calculation. |
| `State` | A State object records a snapshot of the current state of a simulation at a point in time. |

## Forces¶

|  |  |
| --- | --- |
| `AmoebaAngleForce` | This class implements an interaction between triplets of particles that varies with the angle between them. |
| `AmoebaBondForce` | This class implements an interaction between pairs of particles that varies with the distance between them. |
| `AmoebaGeneralizedKirkwoodForce` | This class implements an implicit solvation force using the generalized Kirkwood/Grycuk model. |
| `AmoebaInPlaneAngleForce` | This class implements an interaction at trigonal centers corresponding to the projected in-plane angle bend energy between four particles. |
| `AmoebaMultipoleForce` | This class implements the Amoeba multipole interaction. |
| `AmoebaOutOfPlaneBendForce` | This class implements the Amoeba out-of-plane bend interaction. |
| `AmoebaPiTorsionForce` | This class implements the Amoeba pi-torsion interaction. |
| `AmoebaStretchBendForce` | This class implements the Amoeba stretch-bend interaction. |
| `AmoebaTorsionTorsionForce` | This class implements the Amoeba torsion-torsion interaction. |
| `AmoebaVdwForce` | This class implements a buffered 14-7 potential used to model van der Waals forces. |
| `AmoebaWcaDispersionForce` | This class implements a nonbonded interaction between pairs of particles typically used along with AmoebaGeneralizedKirkwoodForce as part of an implicit solvent model. |
| `AndersenThermostat` | This class uses the Andersen method to maintain constant temperature. |
| `CMAPTorsionForce` | This class implements an interaction between pairs of dihedral angles. |
| `CMMotionRemover` | This class prevents the center of mass of a System from drifting. |
| `CustomAngleForce` | This class implements interactions between sets of three particles that depend on the angle between them. |
| `CustomBondForce` | This class implements bonded interactions between pairs of particles. |
| `CustomCentroidBondForce` | This class is similar to CustomCompoundBondForce, but instead of applying forces between individual particles, it applies them between the centers of groups of particles. |
| `CustomCompoundBondForce` | This class supports a wide variety of bonded interactions. |
| `CustomExternalForce` | This class implements an “external” force on particles. |
| `CustomGBForce` | This class implements complex, multiple stage nonbonded interactions between particles. |
| `CustomHbondForce` | This class supports a wide variety of energy functions used to represent hydrogen bonding. |
| `CustomManyParticleForce` | This class supports a wide variety of nonbonded N-particle interactions, where N is user specified. |
| `CustomNonbondedForce` | This class implements nonbonded interactions between particles. |
| `CustomTorsionForce` | This class implements interactions between sets of four particles that depend on the torsion angle between them. |
| `DrudeForce` | This class implements forces that are specific to Drude oscillators. |
| `Force` | Force objects apply forces to the particles in a System, or alter their behavior in other ways. |
| `GBSAOBCForce` | This class implements an implicit solvation force using the GBSA-OBC model. |
| `HarmonicAngleForce` | This class implements an interaction between groups of three particles that varies harmonically with the angle between them. |
| `HarmonicBondForce` | This class implements an interaction between pairs of particles that varies harmonically with the distance between them. |
| `MonteCarloAnisotropicBarostat` | This class uses a Monte Carlo algorithm to adjust the size of the periodic box, simulating the effect of constant pressure. |
| `MonteCarloBarostat` | This class uses a Monte Carlo algorithm to adjust the size of the periodic box, simulating the effect of constant pressure. |
| `MonteCarloMembraneBarostat` | This is a Monte Carlo barostat designed specifically for membrane simulations. |
| `NonbondedForce` | This class implements nonbonded interactions between particles, including a Coulomb force to represent electrostatics and a Lennard-Jones force to represent van der Waals interactions. |
| `PeriodicTorsionForce` | This class implements an interaction between groups of four particles that varies periodically with the torsion angle between them. |
| `RBTorsionForce` | This class implements an interaction between groups of four particles that varies with the torsion angle between them according to the Ryckaert-Bellemans potential. |
| `RPMDMonteCarloBarostat` | This class is very similar to MonteCarloBarostat, but it is specifically designed for use with RPMDIntegrator. |

## Integrators¶

|  |  |
| --- | --- |
| `AMDForceGroupIntegrator` | AMDForceGroupIntegrator implements a single boost aMD integration algorithm. |
| `AMDIntegrator` | AMDIntegrator implements the aMD integration algorithm. |
| `BrownianIntegrator` | This is an Integrator which simulates a System using Brownian dynamics. |
| `CompoundIntegrator` | This class allows you to use multiple integration algorithms within a single simulation, switching back and forth between them. |
| `CustomIntegrator` | This is an Integrator that can be used to implemented arbitrary, user defined integration algorithms. |
| `DrudeLangevinIntegrator` | This Integrator simulates systems that include Drude particles. |
| `DrudeSCFIntegrator` | This is a leap-frog Verlet Integrator that simulates systems with Drude particles. |
| `DualAMDIntegrator` | DualAMDIntegrator implements a dual boost aMD integration algorithm. |
| `Integrator` | An Integrator defines a method for simulating a System by integrating the equations of motion. |
| `LangevinIntegrator` | This is an Integrator which simulates a System using Langevin dynamics. |
| `MTSIntegrator` | MTSIntegrator implements the rRESPA multiple time step integration algorithm. |
| `RPMDIntegrator` | This is an Integrator which simulates a System using ring polymer molecular dynamics (RPMD). |
| `VariableLangevinIntegrator` | This is an error contolled, variable time step Integrator that simulates a System using Langevin dynamics. |
| `VariableVerletIntegrator` | This is an error contolled, variable time step Integrator that simulates a System using the leap-frog Verlet algorithm. |
| `VerletIntegrator` | This is an Integrator which simulates a System using the leap-frog Verlet algorithm. |

## Extras¶

|  |  |
| --- | --- |
| `Continuous1DFunction` | This is a TabulatedFunction that computes a continuous one dimensional function. |
| `Continuous2DFunction` | This is a TabulatedFunction that computes a continuous two dimensional function. |
| `Continuous3DFunction` | This is a TabulatedFunction that computes a continuous three dimensional function. |
| `Discrete1DFunction` | This is a TabulatedFunction that computes a discrete one dimensional function f(x). |
| `Discrete2DFunction` | This is a TabulatedFunction that computes a discrete two dimensional function f(x,y). |
| `Discrete3DFunction` | This is a TabulatedFunction that computes a discrete three dimensional function f(x,y,z). |
| `LocalCoordinatesSite` | This is a VirtualSite that uses the locations of three other particles to compute a local coordinate system, then places the virtual site at a fixed location in that coordinate system. |
| `LocalEnergyMinimizer` | Given a Context, this class searches for a new set of particle positions that represent a local minimum of the potential energy. |
| `OutOfPlaneSite` | This is a VirtualSite that computes the particle location based on three other particles’ locations. |
| `SerializationNode` | A SerializationNode stores information about an object during serialization or deserialization. |
| `SerializationProxy` | A SerializationProxy is an object that knows how to serialize and deserialize objects of a particular type. |
| `TabulatedFunction` | A TabulatedFunction uses a set of tabulated values to define a mathematical function. |
| `ThreeParticleAverageSite` | This is a VirtualSite that computes the particle location as a weighted average of three other particle’s locations. |
| `TwoParticleAverageSite` | This is a VirtualSite that computes the particle location as a weighted average of two other particle’s locations. |
| `Vec3` | Vec3 is a 3-element tuple that supports many math operations. |
| `VirtualSite` | A VirtualSite describes the rules for computing a particle’s position based on other particles. |
| `XmlSerializer` | XmlSerializer is used for serializing objects as XML, and for reconstructing them again. |

High performance molecular simulation on GPUs


### Navigation

- Application Layer
- Library Layer
  - Core Objects
  - Forces
  - Integrators
  - Extras

©2015, Stanford University and the Authors.
|
Powered by Sphinx 1.3.1
& Alabaster 0.7.7
|
Page source
